# Analysis of six candidate genes as potential modifiers of disease expression in canine XLPRA1, a model for human X-linked retinitis pigmentosa 3

**Published:** 2007-07-11

**Authors:** Richard Guyon, Susan E. Pearce-Kelling, Caroline J. Zeiss, Gregory M. Acland, Gustavo D. Aguirre

**Affiliations:** 1 Section of Ophthalmology, School of Veterinary Medicine, University of Pennsylvania, Philadelphia, PA; 2 James A. Baker Institute for Animal Health, College of Veterinary Medicine, Cornell University, Ithaca, NY; 3 Section of Comparative Medicine, Yale University School of Medicine, New Haven, CT

## Abstract

**Purpose:**

Canine X-linked progressive retinal atrophy (XLPRA) is caused by mutations in *RPGR* exon ORF15, which is also a mutation hotspot in human X-linked retinitis pigmentosa 3 (RP3). The XLPRA1 form of disease has shown extensive phenotypic variability in a colony of dogs that all inherited the same mutant X-chromosome. This variability in onset and severity makes XLPRA1 a valuable model to use to identify genes influencing photoreceptors degeneration in dog and to elucidate molecular mechanisms underlying RP in its human homolog. In this study, *RPGRIP1*, *RANBP2*, *NPM1*, *PDE6D*, *NPHP5*, and *ABCA4* genes were selected on the basis of interaction with RPGR or RPGRIP1 or their implication in related retinal diseases, and were investigated as candidate genetic modifiers of XLPRA1.

**Methods:**

A pedigree derived from an affected male dog outcrossed to unrelated normal mix bred or purebred females was used. Morphologic examination revealed phenotypic variability in the affected dogs characterized as mild, moderate, or severe. Single nucleotide polymorphisms (SNPs) and indel-containing markers spanning the entire genes were designed, based on the canine sequence and the Broad Institute SNP library, and genotyped on the pedigree. For each candidate gene, haplotypes were identified and their frequencies in severely and moderately affected dogs were compared to detect a putative correlation between a gene-specific haplotype(s), and severity level of the disease. Primers were derived from expressed sequence tags (ESTs) and predicted transcripts to assess the relative retinal expression of the six genes of interest in normal and affected retinas of different ages.

**Results:**

Four to seven haplotypes per gene were identified. None of the haplotypes of *RPGRIP1*, *NPM1*, *PDE6D*, *NPHP5*, *RANBP2*, and *ABCA4* were found to co-segregate with the moderate or severe phenotype. No significant difference in the retinal expression levels of the candidate genes was observed between normal and affected dogs.

**Conclusions:**

The haplotype distribution of *RPGRIP1*, *NPM1*, *PDE6D*, *NPHP5*, *RANBP2*, and *ABCA4* suggests these genes are not modifiers of the disease phenotype observed in the XLPRA1 pedigree. The *RPGR*ORF15 stop mutation does not affect the retinal expression of these genes at the mRNA level in the pre-degenerate stage of disease, but no conclusions can be made at this time about changes that may occur at the protein level.

## Introduction

The X-linked retinitis pigmentosa form 3 (RP3), one of the most severe forms of retinitis pigmentosa (RP), is characterized by early onset of central vision loss and night blindness, constriction of visual fields, and complete blindness in young adults [1-5]. Although several genes and genetic loci have been implicated in XLRP, by far the largest proportion of cases results from mutations in the RP GTPase regulator gene (*RPGR*), particularly in the recently characterized exon ORF15 [6,7]. *RPGR* mutations account for about 8-10% of RP cases in North America, and 15-20% in Europe [5,8,9]; of RP simplex patients, about 25% have *RPGR* mutations [2,5,10]. Experimentally produced and naturally occurring models of *RPGR*-XLRP have been described, respectively, for the mouse and dog. In mice, a knockout (KO), resulting from an in-frame deletion of exons 4-6, and a gain of function mutant produced by a truncation of ORF15 have been reported [11,12]. The KO mouse shows normal development and slow degeneration [11], while the gain of function mutant shows early-onset degeneration after abnormal photoreceptor development [12]. In the dog, two microdeletion mutations in exon ORF15 have been identified; a premature stop (XLPRA1) in the Siberian husky and Samoyed breeds, and a frame-shift mutation (XLPRA2) in mixed-breed dogs [13]. The dog is the only naturally occurring model of *RPGR*-XLRP.

In *XLPRA1*, a five nucleotide deletion (del1028-1032) in exon ORF15 causes an immediate premature stop codon that will result in a protein truncated of its 230 C- terminal amino acids. Morphological characterization showed that photoreceptor cells develop and function normally, but then undergo a progressive degeneration of rods and cones. Using morphologic criteria, rods are affected first, while cone degeneration and cell death are later events [13,14]. Because there is normal retinal development before degeneration ensues, the disease is similar to the KO mouse [11], although the degeneration rate comparably is faster. This would suggest that the RPGR function is not essential for normal retinal development, but is required for long-term photoreceptor maintenance and viability.

The pedigree developed to characterize the genetics of the then unknown retinal degeneration locus responsible for XLPRA1, and used to map/clone the disease gene, was constructed from a single affected male outcrossed to unrelated normal females of other breeds that were part of the research colony [14,15]. Even though all affected dogs inherited the single mutant X-chromosome and a stable disease-causing microdeletion, we found that affected males showed variability in onset and severity of the retinal disease phenotype, and these were characterized as mild, moderate, or severe [14]. Due to the breeding strategy used to develop the pedigree, all the affected progeny received the same *RPGR* mutant allele, thus excluding heterogeneity at the primary locus [15]. Environmental factors also were excluded as all dogs were raised in the same environment in a dedicated research colony facility where they received the same diet, medical care, and light exposure. The phenotypic variability observed in XLPRA1, therefore, results from the genetic background, i.e., secondary modifier genes(s), influencing the phenotype. The XLPRA1 dog model represents a valuable resource for the identification of those disease modifier genes that influences the course of *RPGR*-associated photoreceptor degeneration. These modifier genes may lead to the identification of new molecular mechanisms likely to elucidate phenotype variability among human RP3, and possibly among and between other forms of RP.

The means by which mutations in secondary genes influence the effect of a primary mutation has been reviewed [16-18]. Examples include *CYP1B1*, which has been identified as a severity modifier that potentially influences the age of onset of dominant glaucoma caused by *MYOC* mutations [19]. Similarly, the tyrosinase gene has been shown to act as a modifier in glaucoma in the *CYP1B1* KO mouse model [20]. Molecular mechanisms can also rely on interaction of proteins belonging to the same complexes, as exemplified in digenic inheritance in RP caused by concomitant mutations in *RDS* and *ROM1* genes [21].

In this initial screening, we investigated five candidate modifier genes selected on the basis of direct or indirect protein interaction with RPGR or RPGRIP1. These two proteins have been localized to the connecting cilia/proximal outer segment in mice and other mammalian species [22-26]. These genes included *RPGRIP1*, *PDE6D*, *RANBP2*, *NPM1*, and *NPH5*; as a control, *ABCA4* also was evaluated. We tested the simplest hypothesis that any one of these genes acted as a single locus modifier, and determined if the phenotypic variability could be explained by the presence or absence of a specific allele of the gene in homozygous/heterozygous state in dogs divided into different phenotype severity categories. The association between gene, i.e., a specific segregating haplotype, and phenotype was only used as an indicator, but the responsible sequence change could not be inferred if a correlation existed. As the population at our disposal was too small to undertake a linkage analysis, we compared haplotype frequencies in these two phenotype categories. We found no correlation between the degree of disease severity and the candidate genes tested. In parallel, the retinal RNA expression of the candidate genes was evaluated in normal and pre-degenerate mutant retinas. No disease-specific changes in expression were found.

## Methods

### Pedigree resources and disease assessment

Origin and composition of the study colony. All dogs were bred and maintained at the Retinal Disease Studies Facility (RDSF), University of Pennsylvania, New Bolton Center, Kennett Square, PA. These dogs are maintained under specific and standard conditions where all animals have the same exposure to cyclic light (12 h on/12 h off), receive the same diet, and have the same medical procedures and vaccinations. For tissue collection and expression studies, the dogs were anesthetized by intravenous injection of sodium pentobarbital, enucleated, and then euthanized. All procedures involving animals were done in compliance with the ARVO Statement for the Use of Animals in Ophthalmic and Vision Research.

The colony was established by outcrossing one XLPRA1 affected male Siberian husky to unrelated normal female beagles known to be free from inherited retinal degeneration. The carrier and affected progeny was subsequently mated with mixed breed or purebred dogs of varied genetic background, resulting in the creation of a highly polymorphic and informative pedigree [15]. As the initial goal of the project was to produce a sufficient number of informative dogs to fine map the retinal disease, dogs were selected for breeding whose disease phenotype could be readily identified before 1 year of age, or if carrier females were used, would produce more males whose disease status was severe and ascertainment was possible at an early age.

### Determination of the phenotypic status

**Figure 1:**
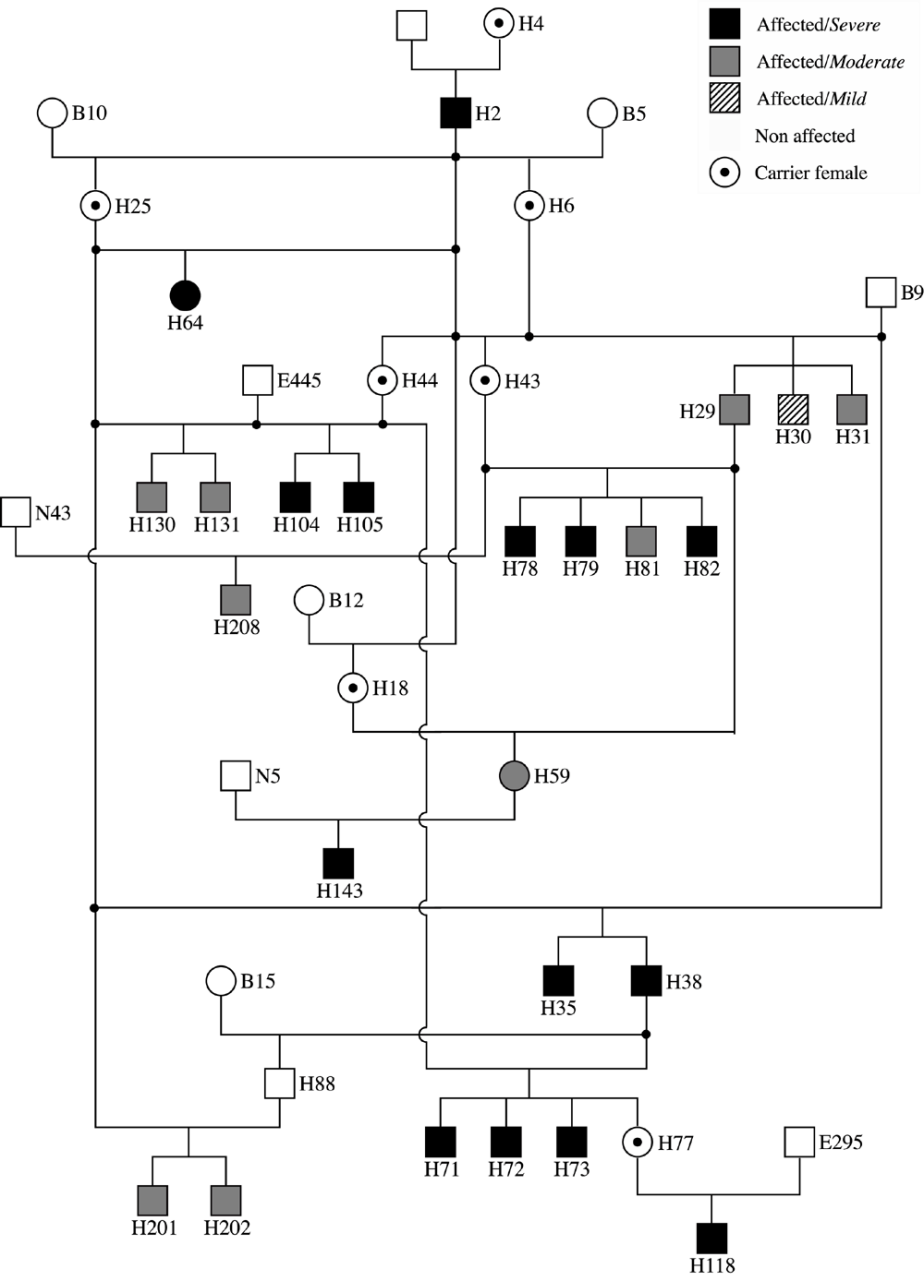
Pedigree of XLPRA1 affected dogs. H2 is a purebred Siberian husky founder that was outcrossed to dogs from various breeds. Those dog breeds contributing to the pedigree include Irish setter, Alaskan malamute, miniature schnauzer, and poodle. Severely affected dogs are in black, moderately affected dogs in dark grey, and mildly affected dogs in crosshatch pattern. Squares represent male and circles represent female. All affected males are hemizygous; affected females H59 and H64 are homozygous for the mutation. Dotted circles are carrier females that were not included in the phenotype analysis. The following abbreviations were used: (B) purebred beagle, (N) mixed breed control, and (E) Norwegian elkhound-derived outcross.

A subset of this colony, consisting of 43 dogs, was selected for the studies (Figure 1); the group included the founder, and some of his descendants. Dogs were included based on the results of serial clinical assessment of retinal disease status using indirect ophthalmoscopy and electroretinography (ERG). Methods for these procedures have been previously described [13,15]. These methods were considered complementary to the main assessment criterion, which was morphologic evaluation using high resolution optical microscopy of 1 μm plastic sections of retinas fixed in paraformaldehyde-glutaraldehyde-osmium tetroxide [27]. Assessment was made with examiner masked as to the genotype (affected, carrier ,and normal), and genotype status of all study dogs was confirmed using a previously described molecular diagnostic test [13]. Included in the study were 22 hemizygous males and 2 homozygous females (Figure 1). The disease characteristics and metrics in both are the same [13,14].

Morphologic criteria were used to establish grades of disease severity taking into account the animal's age, and degree and extent of disease [14]. There were three grades: (1) mild (degeneration present only in periphery after 1.5 years of age or later), (2) moderate (peripheral retinal degeneration develops between 11 and 15 months of age), and (3) severe (photoreceptor degeneration-Stage 2 or more advanced-present both centrally and peripherally) [14]. Of the 24 affected dogs in this subset pedigree, 14 were classified as severe, nine as moderate, and one as mild; In the disease-association studies, only those with moderate and severe disease were used for analysis.

### Sample collection and DNA/RNA extraction

Genomic DNA was isolated from citrated blood or spleen samples either by a standard phenol-chloroform based protocol or by using the QIAamp DNA kit (Qiagen, Valencia, CA) and following the manufacturer's directions. Retinas were collected within 1 min after enucleation under sterile and RNase-free conditions, frozen by immersion in liquid nitrogen, and stored at -70 °C until utilized. Collection of retinal samples was done in the morning to avoid the variations in retinal RNA expression with light:dark cycles [28]. RNA was isolated from retina using a standard guanidinium isothiocyanate-based protocol. For the expression studies, we used 8 affected and 3 controls dogs ranging 8-67 weeks.

### Selection of candidate genes and haplotype identification

Candidate genes: Six genes were chosen for analysis, five of which interact directly or indirectly with RPGR. RPGRIP1 interacts with the N-terminal RCC1-homologous domain of RPGR [25,29]. *NPM1* codes for the chaperone protein that interacts with the C-terminal C2 domain of RPGR [30], NPHP5 is complexed with RPGR and localizes to connecting cilia of photoreceptors [31]. PDE6D interacts with the RCC1-homologous domain of RPGR, and is attached at the disk membranes of rod outer segments [32]. Alternatively, RANBP2 associates with RPGRIP1, and possibly mediates its nucleocytoplasmic shuttling [33]. Though the roles of these proteins in the RPGR complex are not fully understood, numerous mutations have been described leading to retinal degenerations, e.g., Leber congenital amaurosis in the case of *RPGRIP1* [34,35], or Senior-Loken syndrome in the case of *NPHP5* [31]. We selected *ABCA4* as a control gene because of its ubiquitous involvement in other retinal diseases, including Stargardt macular degeneration and RP [36,37].

### Marker selection

Candidate genes were investigated using a fine-scale single nucleotide polymorphism (SNP) analysis [38]. Markers were selected from upstream, downstream, and intronic regions of the genes of interest in an effort to obtain regularly spaced polymorphic markers spanning the entire gene. Polymorphic sites consisting of SNPs and insertion/deletions (indels) were identified in markers designed either based on the Broad Institute SNP database (Broad), or from randomly selected regions picked from the canine sequence (Genome). Markers of length ranging from 300 bp to 1500 bp were designed using Primer3 software (Primer3) using default primers picking conditions (20 bp optimum size, 60 °C optimum Tm). Markers were first genotyped in a subset of the pedigree comprising the most outbred animals in order to check their polymorphism. A minimal set of markers showing informative SNPs or indels was genotyped on the whole pedigree to identify the segregating haplotypes (Table 1). Increasing marker density did not identify more haplotypes because of redundant information provided by markers showing similar pattern of variation.

**Table 1:** Haplotyping marker characteristics and associated polymorphism.

**Name**	**Primer U**	**Primer L**	**Size (bp)**	**Protocol**	**Polymorphism coordinates (1)**		**He (2)**
RPGRIP-5fd2	ATGGCATGAATGCAGTGAAC	CTGGTGTCCTCTGGTCCTGT	518	sequencing (U)	chr15:21,323,467	C/T	0,27
RPGRIP-og	ATGTCTGTGTGTCTATCAGGTG	GTTTGCAGAACACATGGCATTC	305	digestion (Bsu36i)	21 364 923	A/G	0,27
RPGRIP-int2	TTGAATGGTGGGCTAGGAAG	ACCCAAGGCCACTTTACTCA	702	sequencing (U)	21 372 856	A/G	0,43
RPGRIP-3f	GATGAGGTGATGAGGGCCTA	CCGTGGTTAACGTTTGCTTT	1463	digestion (AvaII)	21 434 804	C/T	0,50
RANBP-3fc	TTGGTGAATGCCAAATGAAA	AGCCTGCTGAATGGTTGAAG	717	sequencing (U)	chr10:38,199,076	C/T	0,47
RANBP-int23	ATGCAACAGATGCAAATCCA	CGTTCCTGCCCTTCAAGTAA	1127	sequencing (U)	38 224 775	G/T	0,49
RANBP-int17	GTGGAAACATTCTGGGGAGA	GGGCTTTTTGAATGCTGTGT	1146	sequencing (U)	38 238 339	T/C	0,19
					38 238 358	A/T	0,10
RANBP-int16	TCCCCAATCGCAGAAACTAC	CTCCACCAGGTGTGAATCCT	1197	sequencing (U)	38 242 341	A/C	0,27
RANBP-5f	TTGACATCTTGGGTCCAGTG	TGAATGGGGAAATGATTGCT	1156	digestion (AgeI)	38 272 947	G/T	0,39
				sequencing (L)	38 273 061	A/G	0,19
NPM-snp7	GGCAGAACCCACCTGTAGAA	TTTCTTCGCCCTCAATGTCT	631	sequencing (U)	chr4:43,891,628	A/G	0,27
NPM-3fb	CAGACCCTTAGGCAGACGAG	TTTTTGCAGGCACTTCCTTT	1182	sequencing (U)	43,947,526-7	delAA	0,39
NPM-snp3	TTCTTTGAGCCCATGGAAGT	CTGGCACCCCTCCAAAATA	512	sequencing (U)	43,967,091	C/T	0,49
NPM-5f	GAAATTTGATGGGCAGAGGA	GCCAGGAGCTAGAGGTGATG	1145	digestion (AflIII)	43,971,373-4	insTGTA	0,43
PDE-snp16	TGGTAGGCTGATTTTCTGGTG	CCTGCTTTCCTGGACAAACT	499	sequencing (U)	chr25:46,552,131	A/G	0,27
					46 552 193	C/T	0,19
					46 552 331	C/T	0,10
PDE-snp1	TTTGCATTTCCGAGCTCTTT	ACCAAAACAGGATGGACGAG	511	sequencing (U)	46 577 289	C/T	0,27
PDE-snp10	TTTGGAAAATCAGACGCAAA	GCTTGATCTCGGGGTTATGA	531	sequencing (U)	46 647 231	A/G	0,43
NPHP-snp2	GAAATTAACCCAAACTTCAGCAA	TTCCTTGGCTGTGACTTCCT	464	sequencing (U)	chr33:28,099,052	A/G	0,49
					28 099 062	A/C	0,43
NPHP-snp3b	TTGCATCAACACCTCATTGTC	CCCATCGTTTGATATTCAGAAA	427	sequencing (U)	28 126 211	C/T	0,19
NPHP-snp5b	CTGGCTGATGAGAGGTCTTG	GCTCCTTTTCCTACCTCAACAA	344	sequencing (U)	28 155 878	A/T	0,49
					28 155 919	G/T	0,19
ABCR-snp6	TTACAGGCCTTCCTCCACAC	GGCCAAAGGAAGACACGATA	700	sequencing (U)	chr6:58,159,994	C/T	0,49
					58 160 171	C/T	0,10
					58 160 466	A/G	0,27
ABCR-snp3	TGGTGTTTGGCTTCTGTGAA	CCTTCGGACATGGTTCAGTT	600	sequencing (U)	58 257 868	G/T	0,10
					58 257 905	C/T	0,39

Genotypes were assessed by sequencing PCR products using upper (U) or lower (L) primers, or by digestion with the restriction enzyme indicated in parenthesis. 1. Polymorphism coordinates were based on the version (v2.0) of the canine sequence. 2. Expected heterozygosity (HE) was calculated from the subset of the ten most outbred animals.

### Genotyping protocol and haplotype construction:

PCR reactions were performed on 50 ng of genomic DNA in a final volume of 10 μl containing 0.5 U of AmpliTaq Gold (Applied Biosystems, Foster City, CA) or standard Taq polymerase in 1X of the corresponding reaction buffer, 2 mM MgCl_2_, 250 μM of each dNTP, and 0.3 μM of each primer. All the reactions were carried out in a PTC-200 thermocycler (MJ Research, Waltham, MA) following a "touch-down" program: initial denaturation/induction 95°C, followed by 20 cycles of 30 s at 94 °C, 30 s at 63 °C (decreasing by 0.5 °C per cycle), 1 min at 72 °C, and 15 cycles of 30 s at 94 °C, 30 s at 53 °C, 1 min at 72 °C, and a final extension of 2 min at 72 °C. Hybridization temperatures were lowered by 2 °C when intensity of the signal was too weak.

PCR products were visualized under ultraviolet light with EtBr in 1.8% agarose gels. PCR products were either directly sequenced using upper (U) or lower (L) primer with BigDye chemistry in an ABI capillary sequencer according to standard procedures, or digested by an appropriate restriction enzyme followed by an electrophoresis on an 8% acrylamide gel. Genotyping protocols are shown in Table 1. For each gene, haplotypes were established manually based on the actual and predicted genotypes at every polymorphic site.

### Retinal expression of candidate genes

For each gene of interest, cDNA specific probes were made using either the canine sequence (Genome), dog mRNAs and ESTs when available, and transcript predictions. Probes ranging from 350 to 600 bp were designed with Primer3 software and default conditions. An Aldolase A (*ALDOA*) probe of 151 bp was selected as the internal amplification control [39]. In addition, an opsin probe was used as a control for retinal integrity and, to verify that all samples tested had comparable levels of opsin expression. Reverse transcription was performed using the SuperScript first-strand synthesis system (Invitrogen, Carlsbad, CA) following the manufacturer's procedure. First-strand cDNAs were synthesized starting with 5 μg of total RNA from retina using 50 ng of random hexamer. The cDNAs were purified using the QIAquick kit (Qiagen). In order to estimate the relative amount of a cDNA of interest, we adjust PCR conditions specifically to keep amplification still in the exponential range. PCR reactions were carried out on 20 ng of total cDNA in a 10 μl reaction mix containing the corresponding cDNA marker, 0.12 μM of the *ALDOA* control, 2.5-5 mM MgCl_2_, 0.5 U of AmpliTaq Gold, 1X of reaction buffer, and 250 μM of each dNTP. All reactions were carried out according to the following program: 7 min at 95 °C, followed by 32 cycles of 30 s at 94 °C, 30 s at 63 °C, 1 min at 72 °C and a final extension of 2 min at 72 °C. Primer sequences and specific conditions are described in Table 2.

**Table 2:** Semiquantitative polymerase chain reaction marker characteristics and specific conditions.

**Name**	**Primer U**	**Primer L**	**Size (bp)**	**Primers conc. (μM)**	**MgCl_2_ conc. (mM)**
RPGRIP-rg2	CTGAAGCCAGTGAAGCACAA	TCCACGAGGTCTCCTGATTC	610	0,12	3,5
RANBP-rg2	TTCGAAACAGCTGTCAAGAAAC	CAGGTTTGTCCACAGTTCCA	421	0,12	3,5
NPM-rg1	CAACACATTCTTGGCAATGG	CAGCCAAAAATGCACAAAAA	435	0,12	5
PDE6D-rg1	GGGATGCTGAGACAGGAAAG	CCCAAAACCCAAATTCTTGA	426	0,12	5
NPHP-rg1.2	AGGCCATCTCTCACGGAATA	CTTTCCCTTCTGCCTCCTTC	365	0,6	3
ABCR-rg2	CCATGCTAAGGAAGCTGCTC	GTGGTGTCCCCAGTAAGCAT	490	0,12	2,5
RHO-rg2	CTGTGGTCTTTGGTGGTCCT	AGCAGATCAGGAAAGCGATG	421	0,04	2,5
ALDO-rg3	ATCCTGGCTGCAGATGAGTC	ATAGGATGaCACCCCCAATG	151	0,12	2.5-5

Markers designed from the six genes of interest were amplified together with aldolase gene marker (ALDO) as a control using the indicated conditions. The opsin gene marker (RHO) was amplified in the same manner in order to estimate the retinal integrity. U indicates upper primer; L indicates lower primer.

PCR products were run on a 1.8% agarose gel containing EtBr, and digital images acquired (UV light cabinet; Alpha Innotech Corp., San Leandro, CA). Quantification of the bands was performed with the Alpha Imager software (v4.0) by scanning the lanes and integrating the peaks, thus expressing the band intensity in relative absorbance units. The ratio between the bands corresponding to the cDNA of interest and the internal control was calculated to normalize for initial variations in sample concentration, and as a control for reaction efficiency. The ratios were derived from three independent repeats of the experiments from which the mean, standard deviation and relative standard deviation were calculated.

## Results

### Polymorphism detection

To detect polymorphisms within the pedigree, we picked 19 markers from randomly selected regions of the candidate genes, and 37 markers from the Broad Institute SNP database, and were able to successfully amplify and sequence them in the most outbred dogs of the study pedigree. Both types of markers were chosen from non-coding regions where polymorphism is expected to be the highest. The randomly selected markers were deliberately chosen for their large size in order to screen ample regions of the genes. These markers covered 16.3 kb, and permitted detection of 19 SNPs (1 SNP/850 bp), and 3 indels. Markers based on the SNPs from the Broad Institute were selected so that they would surround multiple SNPs in order to maximize the chance of success. These additional markers covered a total of 20.3 kb, and contained 76 of these putative SNPs. Of these, a total of 47 SNPs (62%) were truly polymorphic after genotyping of the most outbred dogs. Differences in SNP frequencies among the regions investigated were observed; 72% of the putative SNPs in *PDE6D* were polymorphic in the pedigree, but only 40% in *NPM1*. This prompted us to design more markers in regions seemingly less polymorphic. These markers allowed the identification of 16 new SNPs not present in the Broad Institute database, and 8 indels.

Based on their patterns of variation, 21 polymorphic markers designed using both approaches were retained as they make up a minimal set for distinction of haplotypes in the genes of interest. Altogether, these markers spanned 15.9 kb, and included 28 SNPs, and 2 indels (see Table 1). For each polymorphic site, expected heterozygosity (H_E_) was calculated from frequencies of alleles observed in the most outbred animals. Though these values do not reflect the true heterozygosity in the whole population, it was a good estimate of the genetic variability in the pedigree brought by the various contributing breeds. The H_E_ of the polymorphic sites we investigated ranged from 0.10 to 0.50 (Table 1).

### Haplotyping

**Figure 2:**
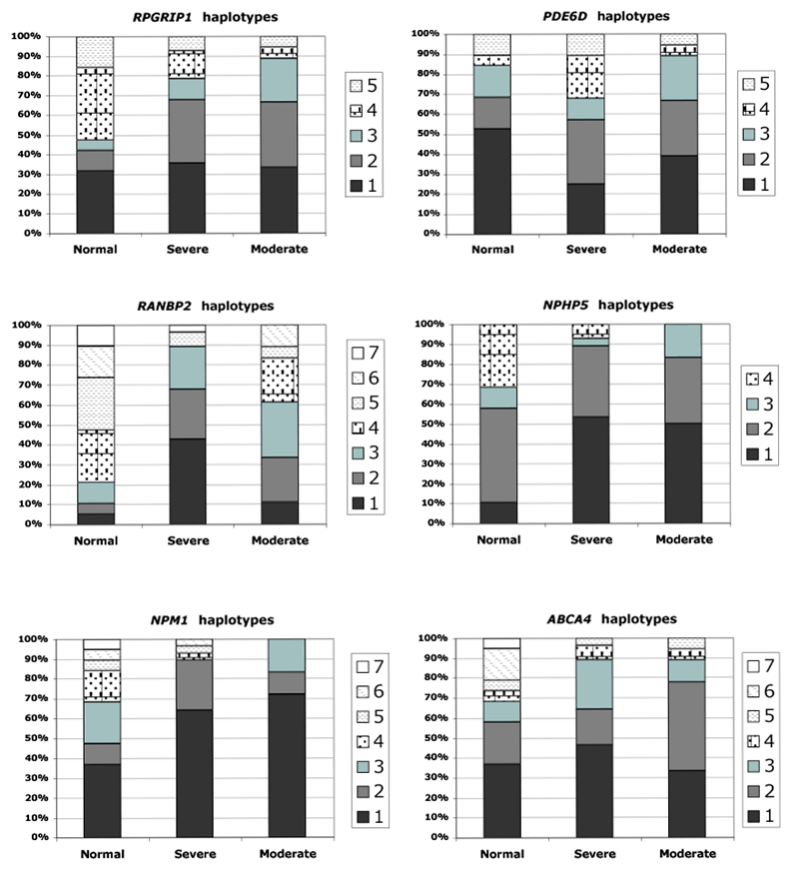
Haplotype frequencies in *RPGRIP1*, *RANBP2*, *NPM1*, *PDE6D*, *NPHP5*, and *ABCA4* in normal dogs, and affected dogs with severe and moderate phenotypes. Haplotypes were numbered from one to seven, and frequencies were calculated from subsets of 10 normal, 9 moderate, and 14 severe XLPRA1 dogs. Table 3 identifies the haplotypes tested for each of the genes.

Two to five markers/gene were selected, and these represented a total of 4-7 polymorphic sites/gene. They delineated genomic regions ranging from 57 kb for *NPHP5* to 111 kb for *RPGRIP1*. The genotyping of these markers allowed the identification of four to seven haplotypes/gene in the normal dogs. The haplotypes of the six genes are presented in Table 3. The genotypes of the dogs are shown in Table 4. The frequencies of the haplotypes of the six genes of interest in affected dogs are presented in Figure 2. The number of haplotypes identified is obviously related to the number of polymorphic sites that define them. The distribution and frequency of these haplotypes in the affected dogs are also consequent to the high level of inbreeding inside the pedigree. In nonaffected dogs, the highest haplotype frequency of about 50% was observed in *PDE6D* and *NPHP5*, while the highest haplotype frequency in *RANBP2* was only 26%. Four out of the seven haplotypes in *NPM1*, *RANBP2*, and *ABCA4* occurred in 10% or less of the chromosomes for the evaluated genes.

**Table 3:** Haplotypes of the six genes of interest tested in the XLPRA1 pedigree.

**RPGRIP1 haplotype**	**1**	**2**	**3**	**4**	**5**		
chr15:21,323,467	C	T	T	C	C		
21,364,923	G	G	G	G	A		
21,372,856	A	G	G	A	G		
21,434,804	C	C	T	T	T		
							
**RANBP2 haplotype**	**1**	**2**	**3**	**4**	**5**	**6**	**7**
chr10:38,199,076	T	T	C	T	C	T	T
38,224,775	T	G	T	G	G	T	T
38,238,339	T	T	C	T	T	T	T
38,238,358	A	T	A	A	A	A	A
38,242,341	A	A	A	A	A	C	A
38,272,947	G	G	G	G	G	T	T
38,273,061	G	G	A	G	G	G	G
							
**NPM1 haplotype**	**1**	**2**	**3**	**4**	**5**	**6**	**7**
chr4:43,891,628	A	G	A	A	A	G	A
43,947,526-7	-	-	+	-	-	-	+
43,967,091	T	T	C	C	T	T	C
43,971,373-4	+	+	-	+	-	-	+
							
**PDE6D haplotype**	**1**	**2**	**3**	**4**	**5**		
chr25:46,552,131	A	A	A	G	G		
46,552,193	C	C	C	C	T		
46,552,331	C	C	C	T	C		
46,577,289	C	C	T	C	C		
46,647,231	G	A	G	A	A		
							
**NPHP5 haplotype**	**1**	**2**	**3**	**4**			
chr33:28,099,052	A	A	G	G			
28,099,062	A	A	A	C			
28,126,211	T	T	C	T			
28,155,878	T	A	A	T			
28,155,919	G	T	T	T			
							
**ABCA4 haplotype**	**1**	**2**	**3**	**4**	**5**	**6**	**7**
chr6:58,159,994	T	T	C	C	C	C	C
58,160,171	T	T	T	C	T	T	T
58,160,466	G	G	G	G	G	A	G
58,257,868	G	G	G	G	T	G	G
58,257,905	T	C	T	T	T	T	C

Polymorphism coordinates were based on the May 2005 version (v2.0) of the canine sequence. Haplotypes are presented in columns and are numbered from one to seven.

**Table 4:** Genotypes of the six genes of interest in severely and moderately affected dogs.

	**RPGRIP1**	**RANBP2**	**NPM1**	**PDE6D**	**NPHP5**	**ABCA4**
**Severely affected**						
H2	2,2	1,2	1,1	1,4	1,1	1,2
H64	2,5	2,1	1,2	4,4	1,2	1,2
H104	1,1	3,2	2,1	5,4	2,1	4,1
H105	1,1	3,1	2,1	5,2	3,1	4,1
H78	3,1	3,1	1,1	1,1	1,1	1,1
H79	3,1	3,1	1,1	1,2	2,1	1,1
H82	1,2	2,1	1,1	1,2	1,1	3,1
H143	4,1	7,1	6,1	1,1	4,1	2,3
H35	3,5	5,1	4,2	2,4	2,1	5,2
H38	4,2	3,1	1,2	2,3	2,2	3,2
H71	4,2	3,2	1,1	2,2	2,1	3,1
H72	2,1	2,1	2,1	3,4	2,1	3,1
H73	2,2	1,1	2,1	3,2	2,1	3,1
H118	1,4	5,2	5,1	5,2	4,2	1,3
**Moderately affected**						
H29	3,1	2,3	1,1	1,2	2,1	3,1
H31	3,2	5,6	1,1	1,2	2,3	5,1
H130	1,2	3,4	1,1	3,3	3,1	4,2
H131	4,5	3,4	1,2	5,3	3,1	1,2
H81	3,2	3,2	1,1	1,2	2,1	1,1
H208	1,1	4,2	3,1	1,2	2,1	1,2
H59	3,1	2,1	1,3	1,2	1,1	3,2
H201	2,2	6,1	3,1	1,4	2,1	2,2
H202	1,2	3,4	1,2	1,3	2,1	2,2

Alleles are numbered from one to seven according to haplotypes shown in Table 3. Genotypes that are present in both severe and moderate phenotypes are in red color.

When we compared the occurrence of genotypes in severely and moderately affected dogs, most of the genotypes actually were present in both groups (Table 4). When looking at specific haplotype frequencies in severe versus moderately affected dogs, we observed no significant differences in the major haplotypes for *RPGRIP1*, *NPM1*, *NPHP5*, *ABCA4*, and *PDE6D*. Frequencies between the two phenotypes were similar in the case of *RPGRIP1*, *NPM1*, and *NPHP5* major haplotypes, and not discrepant enough in *ABCA4* and *PDE6D* to account for the level of affection. By contrast, a discrepancy was observed in the *RANBP2* haplotype 1 frequency between severely and moderately affected dogs (43% versus 11%). This haplotype was present in 11 out of 14 severely affected dogs (heterozygous in 10, and homozygous in 1), but only in two out of nine moderately affected dogs. In addition, 15 haplotypes were considered as minor in the affected dogs, i.e. accounting for five chromosomes or fewer out of the 46 chromosomes for the gene considered.

### Gene expression

**Figure 3:**
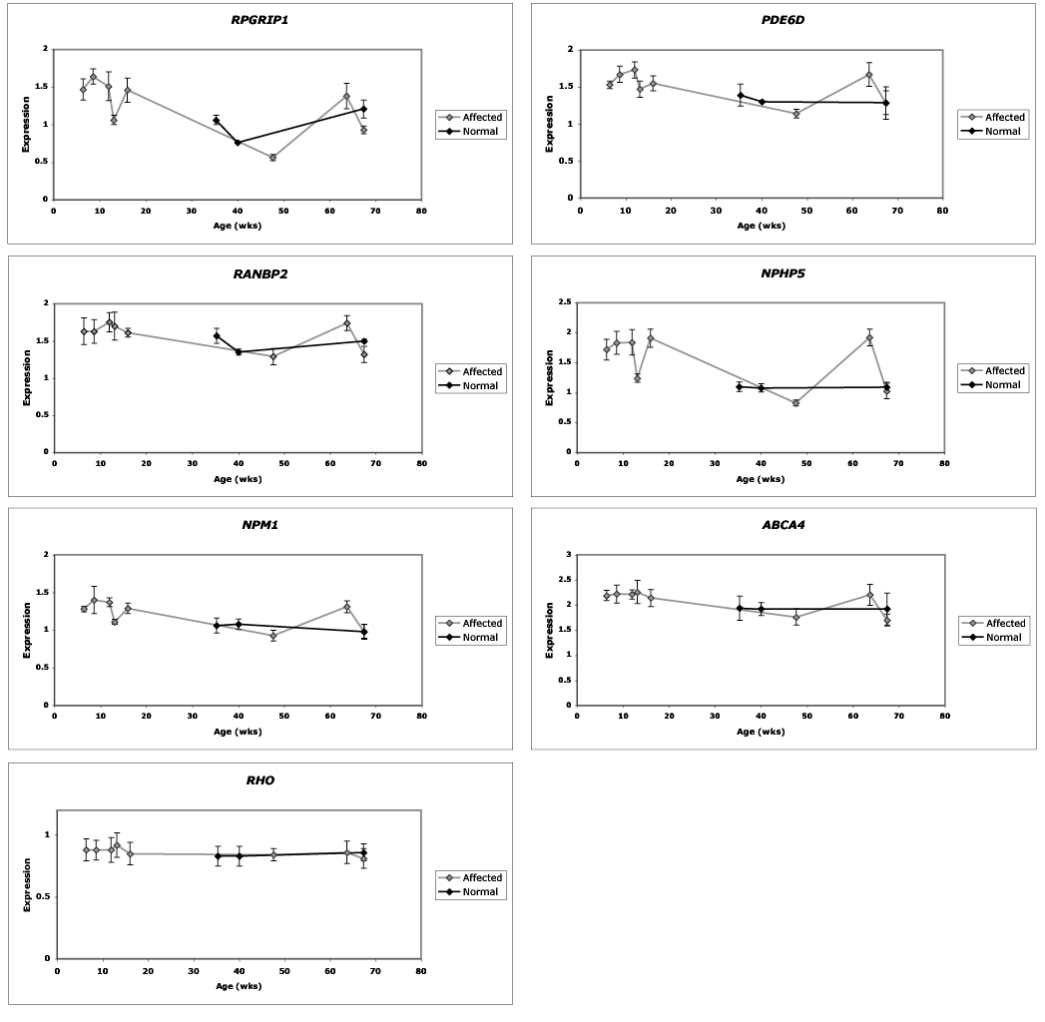
Relative expression levels of *RPGRIP1*, *RANBP2*, *NPM1*, *PDE6D*, *NPHP5*, and *ABCA4* genes in pre-degenerate XLPRA1 affected retinas. Expression level of opsin (RHO) is also shown as a control of retinal integrity. Normal retinas are in black symbols and lines; affected retinas are in light gray symbols and lines. Standard deviations calculated from 3 repeats also are shown.

Expression studies were done on total retinal RNA from eight affected and three nonaffected dogs to examine the relative expression level of the candidate genes. This was determined by amplifying cDNA-specific markers and reporting PCR products relative to the *ALDOA* control. By this method, only the quantitative aspect of transcript levels were estimated, but no conclusions can be made at this time about changes that may occur at the protein level. The results were highly reproducible as evidenced by the relative standard deviation (SD/AV) calculated from the three independent repeats. In most cases, this ratio did not exceed 10% for a given gene in a given retina. Relative expression as a function of age in affected and nonaffected dogs for the six genes of interest is presented on Figure 3.

To assess the retinal integrity, we first examined opsin expression to establish the integrity of the retinal samples, and found no differences between control and mutant retinas. *RANBP2*, *NPM1*, *PDE6D*, and *ABCA4* showed a rather constant expression in affected retinas, and the expression did not exceed a 1.5 fold difference in relative units from 6 to 67 weeks of age. Control retinas showed slight variations for the period considered. Therefore age or status does not have any notable effect on these genes for the time period analyzed. In contrast, *NPHP5* expression varied by a more than twofold difference in relative units from 6 to 67 weeks of age while the control retinas showed a steady expression. Similarly, *RPGRIP1* expression varied by a threefold difference in relative units. In this case, however, the control retinas also showed some fluctuations for the time period examined. These variations are obviously not related to the age, and their relationship with the affected status is unknown at this time. As the expression in the affected retinas showed a similar pattern that differed primarily in magnitude for some of the genes, it is likely that these differences reflect specific sample differences rather than a disease-specific change.

## Discussion

The canine XLPRA1 model of *RPGR*-XLRP is a naturally occurring microdeletion that results in a stop mutation in exon ORF15, the mutation hotspot for *RPGR* in humans [13]. In the study population, the disease originated from a single mutant X-chromosome, yet affected animals showed great phenotypic variability in severity. As the disease represents a stable mutation with no heterogeneity at the primary locus (*RPGR*), the XLPRA1 pedigree is a suitable resource to examine genetic modifiers on the phenotypic variability of disease. Such studies would have direct relevance to human XLRP.

However the search for such genetic modifiers of phenotype by classical association methods is difficult due to the very characteristics of that trait of interest, and limitations in the study population. Because the severity status is accessible only in affected animals, matings with normal or carrier dogs are not fully informative on the transmission of the phenotype, preventing a classical linkage analysis. Linkage disequilibrium (LD) analysis would provide an alternative approach to identifying shared haplotypes correlated with a phenotype. This was successfully carried out in dogs recently, and enabled an ancestral disease transmitting chromosome with a novel retinal degeneration gene to be identified [38,40]. This approach would require multiple independently ascertained affected individuals that are not closely related. However, XLPRA1 does not exist outside the specific research colony as commercial testing over a 3-4 year period has not identified any affected individuals or mutant chromosomes in the population (Dr. Jeanette Felix, *OptiGen LLC*, personal communication). Thus the relatively small and inbred affected population from our pedigree is not suitable for analysis using LD.

Because of these limitations, we directed our analysis to identifying candidate gene-specific haplotypes, and their frequencies were compared in severely versus moderately affected dogs to determine if there were a putative correlation between haplotype and phenotype. Such an approach is now possible given the recent wealth of canine genomic resources, e.g. 1.5x TIGR sequence [41], 7.6x public sequence [42], and a robust SNP resource (Broad). In this study we investigated six candidate modifier genes. *RPGRIP*, *RANBP2*, *NPM1*, *PDE6D*, and *NPHP5* were selected on the basis of protein interaction with RPGR or RPGRIP1 as these two proteins independently cause photoreceptor degeneration when mutated; *ABCA4* was selected as a control gene, and because of its association with macular degeneration and RP [36,37].

By carrying out this strategy, some of the characteristics of the XLPRA1 pedigree were revealed. The random sequencing of 16.3 kb of non-coding regions around *RPGRIP1*, *RANBP2*, and *NPM1* genes gave us an estimated SNP discovery rate of 1/850 bp. Even though the outcrossed pedigree represents founders from multiple breeds, e.g. beagle, elkhound, and Irish setter in addition to Siberian husky [15], which would have been expected to contribute genotypic diversity and breed-specific polymorphisms, this figure is in close agreement to the 1/1000 bp reported for the dog genome [42]. Moreover, the Siberian husky, like the Alaskan malamute, belong to the Asian breed cluster [43], and is expected to diverge even more from the other contributing breeds. Therefore the polymorphism in our pedigree is mainly of inter-breed origin as a small number of founder in each breed were used.

The number of haplotypes finally identified is clearly related to the number of polymorphic sites used to define them. Although we identified additional polymorphisms, these brought redundant information, and are not included in the analysis. Because of the structure of our pedigree, we estimated that a maximum of 13 of the 20 chromosomes brought by the 10 founders could have contributed to the affected dogs. However, only four to seven haplotypes were identified in each of the candidate modifier genes analyzed. This discrepancy arises, in part, by the high inbreeding of the pedigree, and is also a function of the gene locus analyzed. In most dog breeds, the genome features regions of near-total homozygosity alternating with regions of high heterozygosity, implying that the genome is comprised of large blocks with limited diversity [42,44]. Thereby the haplotype diversity of a given region in our pedigree depends on the heterozygosity of that region within and between the contributing breeds.

We found that moderately and severely affected dogs had no major discrepancies in haplotype frequencies for *RPGRIP1*, *NPM1*, *PDE6D*, *NPHP5*, and *ABCA4*, which suggests these genes are not modifiers of disease phenotype. In contrast, we found an overrepresentation of *RANBP2* haplotype 1 in the severe disease class, with 11 of the 14 dogs in the group having this haplotype in one [10] or both [1] chromosomes. This distribution would indicate that the *RANBP2* haplotype 1 behaves as a susceptibility allele for the disease. On closer examination, however, if we exclude the dog H2, the severely affected group consists of nine dogs with only one parent heterozygous for the haplotype 1 i.e. 50% chance to inherit this allele, and four dogs with both parents heterozygous, i.e. 75% chance to inherit at least one copy of haplotype 1. The moderately affected group consists of seven dogs with only one parent heterozygous for the haplotype 1, i.e. 50% chance to inherit the haplotype 1 and two dogs with no parents with this haplotype. Therefore the observed ratio of 10 severely affected dogs with the haplotype 1 versus two moderately affected dogs is not significantly different from what was expected (7.5 versus 3.5), and an association between *RANBP2* and disease severity can not be made.

In a complementary study, we analyzed the relative expression of the candidate modifier genes in normal and pre-degenerate XLPRA1 retinas in relation to an internal control gene [39]. Because severity status could not be determined a priori in the samples used for the expression studies, we selected ages prior to photoreceptor degeneration, and used opsin expression as an independent measure of retinal integrity. Both the normal and mutant samples showed the same levels of opsin expression, thus establishing that the photoreceptors were not degenerate. Furthermore, we reasoned that if there were differential expression of the candidate genes between severely and moderately affected retinas, a difference between normal and affected retina should be identified first. In the case of *RANBP2*, *NPM1*, *PDE6D*, and *ABCA4*, the relative expression levels of affected and non-affected retinas were comparable for the time period analyzed. The slight decrease in expression level of affected retinas between 20 and 50 weeks may reflect that this large time interval contains only a single sample that may not be representative. Other than that, most of variations between normal and affected retinas did not exceed a twofold difference, which was considered not large enough to be of significance. For *RPGRIP1* and *NPHP5*, however, a lower expression was observed in 48 (*RPGRIP1*, *NPHP5*), and 67 (*RPGRIP1*) week old affected retinas that could reflect initial stages of the disease process, prior to the onset of degeneration. However, these values are similar to those obtained from the control retinas. This will require examination of additional samples at different time points using more quantitative approaches, and would be facilitated by having homogeneous lines of dogs that predictably have moderate or severe disease.

In this study, we used a semi quantitative method to examine variations in mRNA expression levels between normal and affected retinas. Qualitative variations at the sequence level in the candidate genes may also exist between normal and affected retinas. As well, variations at the protein level could exist, and possibly be responsible for the observed phenotype variations. Additional studies would be required to address these issues.

In addition to the genes analyzed, other genes have been identified recently that interact with *RPGR* or *RPGRIP*, and warrant further examination. With expansion of the pedigree resources currently in progress, these genes will be examined in a subsequent screen. *NPHP4* codes for an RPGRIP-interacting protein [45], and *NPHP6* for an RPGR associated protein [46]; both are good candidate modifier genes as, when mutated, they result in retinal degeneration. In addition, SMC1 and SMC3 have been shown to interact directly with RPGR via its RCC1 domain, while IFT88 and kinesin-2 proteins have been shown to be part of the RPGR complex [47]. Candidate modifier genes also could be selected following other criteria, but one of the most promising ways to identify the genes is to investigate the genome in its entirety. Such studies have already been performed in mice by mating strains showing variable loss of photoreceptors in age-related retinal degeneration [48], or after prolonged light exposure [49]. This led to the identification of quantitative trait loci (QTLs) with protective alleles in both cases. The resources needed to do this work in dogs are not available at present, or in the foreseeable future. However, once candidate genes are identified they can be readily tested in association studies using the available pedigree resources.
